# The Biosynthesis of Lipooligosaccharide from *Bacteroides thetaiotaomicron*

**DOI:** 10.1128/mBio.02289-17

**Published:** 2018-03-13

**Authors:** Amy N. Jacobson, Biswa P. Choudhury, Michael A. Fischbach

**Affiliations:** a Department of Bioengineering and Therapeutic Sciences and California Institute for Quantitative Biosciences, University of California, San Francisco, California, USA; b Chemistry and Chemical Biology Graduate Program, University of California, San Francisco, California, USA; c GlycoAnalytics Core, University of California, San Diego, California, USA; VA Palo Alto Health Care System

**Keywords:** *Bacteroides*, lipopolysaccharide, microbiome

## Abstract

Lipopolysaccharide (LPS), a cell-associated glycolipid that makes up the outer leaflet of the outer membrane of Gram-negative bacteria, is a canonical mediator of microbe-host interactions. The most prevalent Gram-negative gut bacterial taxon, *Bacteroides*, makes up around 50% of the cells in a typical Western gut; these cells harbor ~300 mg of LPS, making it one of the highest-abundance molecules in the intestine. As a starting point for understanding the biological function of *Bacteroides* LPS, we have identified genes in *Bacteroides thetaiotaomicron* VPI 5482 involved in the biosynthesis of its lipid A core and glycan, generated mutants that elaborate altered forms of LPS, and used matrix-assisted laser desorption ionization–time of flight (MALDI-TOF) mass spectrometry to interrogate the molecular features of these variants. We demonstrate, *inter alia*, that the glycan does not appear to have a repeating unit, and so this strain produces lipooligosaccharide (LOS) rather than LPS. This result contrasts with *Bacteroides vulgatus* ATCC 8482, which by SDS-PAGE analysis appears to produce LPS with a repeating unit. Additionally, our identification of the *B. thetaiotaomicron* LOS oligosaccharide gene cluster allowed us to identify similar clusters in other *Bacteroides* species. Our work lays the foundation for developing a structure-function relationship for *Bacteroides* LPS/LOS in the context of host colonization.

## IMPORTANCE

Much is known about the bacterial species and genes that make up the human microbiome, but remarkably little is known about the molecular mechanisms through which the microbiota influences host biology. A well-known mechanism by which bacteria influence the host centers around lipopolysaccharide (LPS), a component of the Gram-negative bacterial outer membrane. Pathogen-derived LPS is a potent ligand for host receptor Toll-like receptor 4, which plays an important role in sensing bacteria as part of the innate immune response. Puzzlingly, the most common genus of human gut bacteria, *Bacteroides*, produces LPS but does not elicit a potent proinflammatory response. Previous work showing that *Bacteroides* LPS differs structurally from pathogen-derived LPS suggested the outlines of an explanation. Here, we take the next step, elucidating the biosynthetic pathway for *Bacteroides* LPS and generating mutants in the process that will be of great use in understanding how this molecule modulates the host immune response.

## INTRODUCTION

High-abundance cell-associated molecules are of great interest to understanding microbiota-host interactions at the level of molecular mechanism. Unlike high-abundance diffusible molecules, which are the characteristic products of amino acid and sugar metabolism, high-abundance cell-associated molecules are often lipids and glycolipids (1). These molecules tend to be structural components of the cell membrane or cell wall that are architecturally similar although chemically different among bacterial taxa—lipopolysaccharides (LPSs), lipoteichoic and wall teichoic acids, mycolic acids, and muramyl dipeptides are key examples. Their ubiquity on the cell surface makes them excellent targets for bacterial detection by innate immune receptors, including Toll-like receptors and NOD proteins (2). However, a longstanding question remains: how do innate immune cells “know” whether the bacterial cell that they encounter is a mutualist or a pathogen and “decide” how to respond appropriately? Part of the answer likely involves unique strain-specific chemical signatures within these cell-associated molecules.

Lipopolysaccharide (LPS) is a canonical cell-associated glycolipid. The interaction between LPS and host Toll-like receptor 4 (TLR4) is a paradigm for immunologic sensing of Gram-negative bacteria. LPS is generally composed of a lipid anchor (termed lipid A), a core oligosaccharide region, and a polysaccharide repeating unit called the O antigen. The core oligosaccharide and O antigen are typically biosynthesized from separate gene clusters, while lipid A biosynthetic genes are distributed throughout the genome (3). The chemical structure of LPS varies considerably among species, and these differences in structure are relevant to function. For example, *Yersinia pestis* deacylates its lipid A when infecting humans, thus avoiding detection by TLR4 (4). *Helicobacter pylori* elaborates its O antigen with Lewis antigens to mimic host glycans (5, 6). More drastic changes in overall structure have also been observed. Species of *Neisseria* produce an LPS variant, known as lipooligosaccharide (LOS), which has a more elaborate core oligosaccharide in place of the conventional O antigen (7, 8). Notably, almost everything known about the biosynthesis, structure, and function of LPS comes from studies of “conventional” pathogens. Remarkably little is known about LPS from commensal organisms and its importance to host innate immunity.

Among the glycolipids found in the gut microbiome, *Bacteroides* LPS is of particular interest. *Bacteroides* and, in ~10% of humans, its relative *Prevotella* are the only high-abundance Gram-negative bacterial genera in the gut. *Bacteroides* as a genus makes up ~50% of the typical Western gut community (9). Notably, the species distribution within that 50% is highly variable between individuals (10). Different *Bacteroides* species have been reported to produce LPS molecules with distinct architectures based on their banding pattern on an SDS-PAGE gel, suggesting that each *Bacteroides* species has the potential to influence innate immunity in its own way (11, 12). *Bacteroides* LPS is already known to have a different lipid A structure than “pathogenic” LPS: *Bacteroides thetaiotaomicron*, *Bacteroides fragilis*, and *Bacteroides dorei* produce penta-acylated, monophosphorylated lipid A, in contrast to the hexa-acylated, diphosphorylated lipid A from *Escherichia coli* (13–16). With a recent exception reporting a *B. thetaiotaomicron* lipid A phosphatase, very little is known about the biosynthetic genes involved in *Bacteroides* LPS biogenesis (17).

It takes as little as 50 ng of *E. coli* LPS injected intravenously into a mouse to cause septic shock (18). In contrast, given our laboratory purification yield of approximately 10 mg *B. thetaiotaomicron* LPS per 1 liter of confluent culture and assuming ~7 × 10^11^ bacteria per liter *in vitro* and 20 trillion *Bacteroides* cells per individual, we estimate that a typical Western human gut contains ~300 mg of *Bacteroides* LPS, likely making it one of the highest-abundance bacterially derived molecules present (19). We set out to better define the biosynthesis and structure of *Bacteroides* LPS as a starting point for understanding and manipulating the interaction between *Bacteroides* and the mammalian immune system.

## RESULTS AND DISCUSSION

### Characterization of the *Bacteroides* lipid A core.

**FIG 1:**
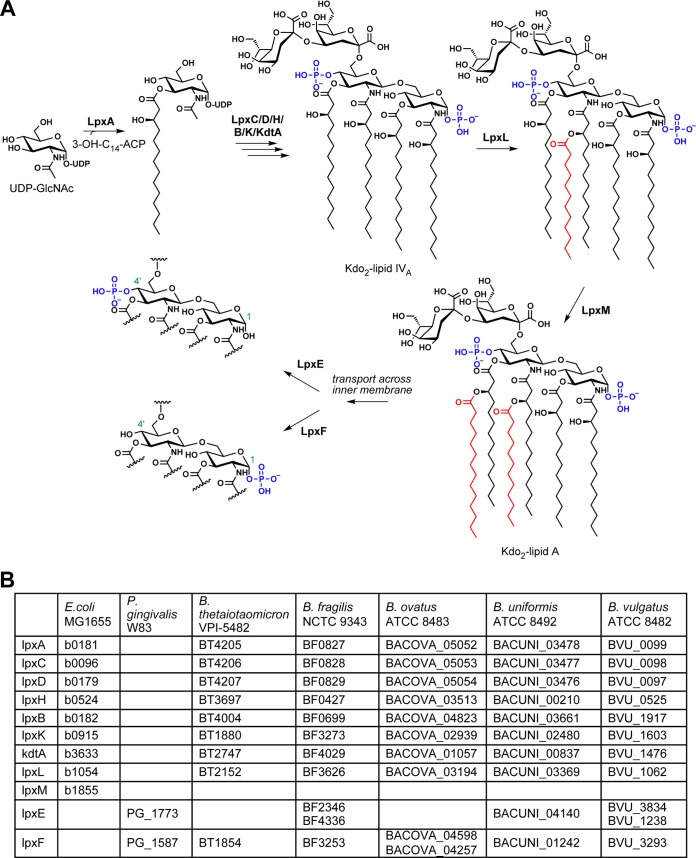
The Raetz pathway for lipid A biosynthesis in *E. coli* and its homologs in *Bacteroides*. (A) Abbreviated schematic of the Raetz pathway, where starting material UDP-GlcNAc is acylated, glycosylated, and phosphorylated by a series of nine biosynthetic enzymes to produce 3-deoxy-d-manno-octulosonic acid 2 (Kdo_2_)-lipid A. *E*. *coli* produces lipid A that is phosphorylated at both the 1 and 4′ positions on the diglucosamine backbone, but the lipid A 1- and 4′-phosphatases LpxE and LpxF have been identified in other bacteria and are thought to act after biosynthesis of Kdo_2_-lipid A is complete. (B) Locus tags of homologs of the Raetz pathway genes in *E. coli* MG1655 and *lpxE* and *lpxF* in *Porphyromonas gingivalis* W83 from a selection of *Bacteroides* species. *Bacteroides* has homologs for every gene in the pathway except that it has only one secondary acyltransferase, suggesting that its lipid A is predominantly penta-acylated. The species vary more in their putative homologs of the *P. gingivalis* phosphatases.

In order to identify candidate biosynthetic genes for *Bacteroides* lipid A, we performed BLAST searches against *Bacteroides* genomes using, as queries, the *E. coli* MG1655 lipid A biosynthesis genes (20). *E. coli* normally produces a lipid A molecule that has six acyl chains and two phosphate groups, as shown in Fig. 1A labeled “Kdo_2_-lipid A.” As expected, orthologs of each Raetz pathway enzyme were identified, except that the *Bacteroides* species had only one ortholog of the acyltransferases LpxL and LpxM; we refer to this ortholog as LpxL for simplicity. LpxL and LpxM are responsible for adding the fifth and sixth acyl chains to *E. coli* lipid A, so the presence of only one of these acyltransferases in *Bacteroides* genomes is consistent with published reports that *B. thetaiotaomicron*, *B. fragilis*, and *Bacteroides dorei* lipid A is penta-acylated rather than hexa-acylated (13–15). *Bacteroides vulgatus* was the only surveyed species to have a second LpxL/LpxM homolog, BVU_1014. Previous work indicates that this gene is part of an aryl polyene gene cluster, indicating that it likely transfers an acyl chain to a non-LPS substrate (21). We next used BLAST to predict lipid A phosphorylation by sequence homology to the lipid A 1- and 4′-phosphatases discovered in *Porphyromonas gingivalis* (22, 23) (Fig. 1). While this search resulted in only one candidate for some species like *B. thetaiotaomicron*, for others there were multiple candidates, and experimental validation will be necessary to conclude which, if any, perform the predicted function.

**FIG 2:**
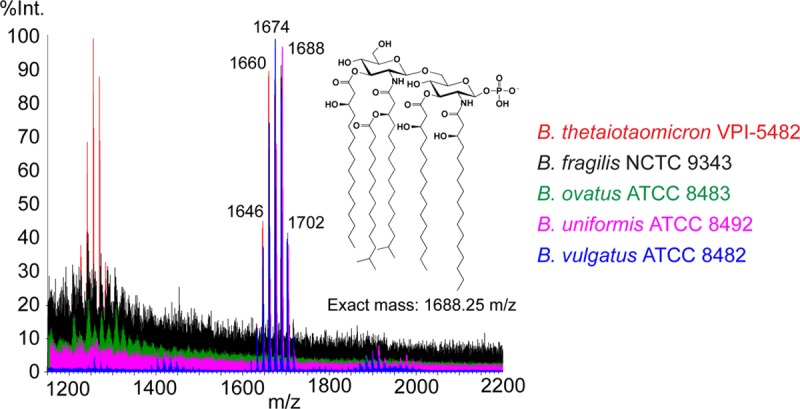
MALDI-TOF MS analysis of lipid A isolated from five *Bacteroides* species. Lipid A was purified from *B. thetaiotaomicron* VPI 5482, *B. fragilis* NCTC 9343, *B. ovatus* ATCC 8343, *B. uniformis* ATCC 8492, and *B. vulgatus* ATCC 8482 by the TRI reagent method, dissolved in 3:1 chloroform-methanol, spotted on a 5-chloro-2-mercaptobenzothiazole (CMBT) matrix, and analyzed on a Waters Corporation Synapt G2 HDMS 32k MALDI-TOF instrument in reflectron negative-ion mode. All five have as their dominant lipid A species a cluster of peaks around 1,688 *m/z* corresponding to the published structure of *B. thetaiotaomicron* lipid A. The peaks in the cluster are separated by 14 *m/z* (methylene group, CH_2_), likely caused by heterogeneity in the number of carbons in each acyl chain of lipid A.

In order to determine the lipid A profile of each species, we isolated lipid A from five common *Bacteroides* species using the TRI reagent method and characterized their lipid A profile by matrix-assisted laser desorption ionization–time of flight (MALDI-TOF) mass spectrometry (MS) (24). Consistent with previous reports, the structures of *B. thetaiotaomicron* VPI 5482 and *B. fragilis* NCTC 9343 lipid A are penta-acylated and monophosphorylated, with their MALDI spectra showing a cluster of peaks around 1,688 *m/z* (13, 14). Moreover, *Bacteroides uniformis* ATCC 8492, *B. vulgatus* ATCC 8482, and *Bacteroides ovatus* ATCC 8483 produce lipid A with virtually identical mass spectra (Fig. 2). Because the bacteria were grown in rich medium under normal anaerobic growth conditions, we cannot be certain that the structure of their lipid A is the same under conditions of host colonization, nor do we know whether it can change in response to stresses encountered in the host.

### Characterization of late biosynthetic genes in *B. thetaiotaomicron* lipid A biosynthesis: acylation and dephosphorylation.

**FIG 3:**
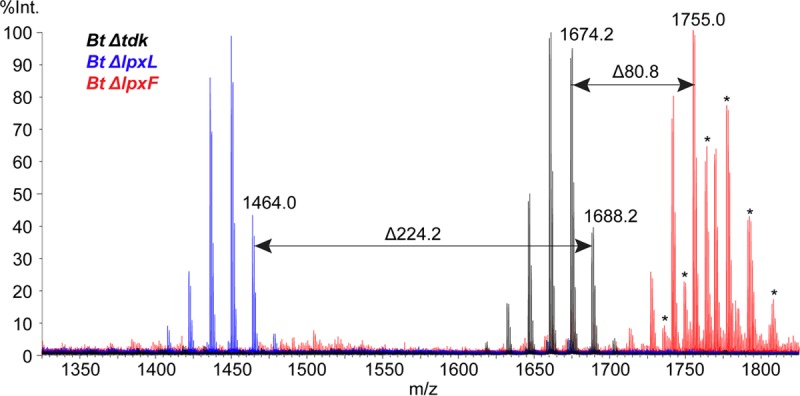
MALDI-TOF mass spectra of lipid A from *B. thetaiotaomicron* lipid A mutants. Lipid A was isolated from *B. thetaiotaomicron* Δ*lpxL* and *B. thetaiotaomicron* Δ*lpxF* strains by the TRI reagent method, dissolved in 3:1 chloroform-methanol, spotted on a CMBT matrix, and analyzed on a Waters Corporation Synapt G2 HDMS 32k MALDI-TOF instrument in reflectron negative-ion mode. The *B. thetaiotaomicron* Δ*lpxL* strain produced a spectrum where the cluster of peaks characteristic of lipid A has decreased in mass by 224 Daltons, the same mass as the 15-carbon secondary acyl chain from the published *B. thetaiotaomicron* structure. The *B. thetaiotaomicron* Δ*lpxF* strain lipid A increased by 80 Daltons over that in wild-type *B. thetaiotaomicron*, indicating the presence of a phosphate group as expected. Sodiated peaks are marked with an asterisk.

Because lipid A is typically an essential component of the outer membrane of Gram-negative bacteria, deletion of genes in the lipid A biosynthetic pathway is frequently lethal to bacteria (25–30). Interestingly, the later biosynthetic genes such as those for the acyltransferases (*lpxL* and *lpxM*) and phosphatases (*lpxE* and *lpxF*) can often be deleted (31). Working in *B. thetaiotaomicron* VPI 5482 *Δtdk*, our background strain for genetic knockouts lacking the thymidine kinase gene *tdk*, we made scarless single deletions of the putative *lpxL* and *lpxF* orthologs that we had identified by BLAST search (BT2152 and BT1854, respectively) and isolated lipid A from the resulting mutants. MALDI-TOF analysis showed a loss of 224 Daltons in the Δ*lpxL* mutant, consistent with the loss of a 15-carbon acyl chain, and a gain of 80 Daltons in the Δ*lpxF* mutant, indicating the addition of a phosphate group (Fig. 3). The assignment of BT1854 as the *B. thetaiotaomicron* lipid A 4′-phosphatase represents independent confirmation of a result first reported by Goodman and coworkers (17).

Lipid A acylation and phosphorylation are important both for bacterial membrane physiology and for the interaction between LPS and host TLR4. An Δ*lpxL* Δ*lpxM* double mutant of *E. coli* MG1655—which lacks the fifth and sixth acyl chains on lipid A, yielding a tetra-acylated, diphosphorylated molecule referred to as lipid IV_A_—cannot grow above 32°C (Fig. 1A) (32). The crystal structure of the TLR4/MD-2 complex with *E. coli* lipid A suggests that the number of acyl chains and the number and position of phosphate groups on the molecule may affect binding affinity to the receptor and possibly receptor dimerization (33). *E. coli* lipid IV_A_ is capable of inhibiting TLR4 activation by wild-type *E. coli* lipid A (34). This tetra-acylated diphosphorylated scaffold has been used in the design of eritoran, a lipid A mimic developed as a TLR4 antagonist as a potential treatment for sepsis (35). Additionally, lipid A mutants in *E. coli* differentially stimulate NF-κB production in a THP-1 reporter cell line (36). We hypothesize that identifying lipid A biosynthesis genes in *Bacteroides* will allow us to make mutants that may have different immunostimulatory abilities and could be used to control innate immune responses in a host. Toward this goal, we have generated a *B. thetaiotaomicron* Δ*lpxL* Δ*lpxF* double mutant that elaborates tetra-acylated, diphosphorylated lipid A, which we anticipate will be a TLR4 antagonist (see Fig. S1 in the supplemental material).

10.1128/mBio.02289-17.1 FIG S1  The double knockout of *lpxL* and *lpxF* in *B. thetaiotaomicron* produces tetra-acylated diphosphorylated lipid A. Lipid A was isolated from the *B. thetaiotaomicron ΔlpxL ΔlpxF* strain using the TRI reagent method. The resulting material was dissolved in 3:1 chloroform-methanol, mixed in a 1:1 ratio with saturated 5-chloro-2-mercaptobenzothiazole in 3:1 chloroform-methanol, and spotted on a Waters Corporation MALDI target. The sample was analyzed on a Waters Synapt G2 MALDI-TOF mass spectrometer in reflectron negative-ion mode. The mass of the resulting cluster of peaks is around 1,544 *m/z*, which is 144 *m/z* less than wild-type *B. thetaiotaomicron* lipid A at 1,688 *m/z*. That 144 Daltons decrease is equivalent to the loss of 224 Daltons observed in the *B. thetaiotaomicron ΔlpxL* mutant plus the addition of 80 Daltons as observed in the *B. thetaiotaomicron ΔlpxF* mutant, indicating that we have made a tetra-acylated diphosphorylated lipid A molecule in *B. thetaiotaomicron*. *B. thetaiotaomicron ΔlpxL ΔlpxF* strain peaks are accented in blue for clarity, and sodiated *B. thetaiotaomicron ΔlpxL ΔlpxF* strain peaks are in black and marked with an asterisk. Download FIG S1, EPS file, 2.3 MB. Copyright © 2018 Jacobson et al. 2018 Jacobson et al. This content is distributed under the terms of the Creative Commons Attribution 4.0 International license.

### SDS-PAGE analysis of *Bacteroides* LPS.

**FIG 4:**
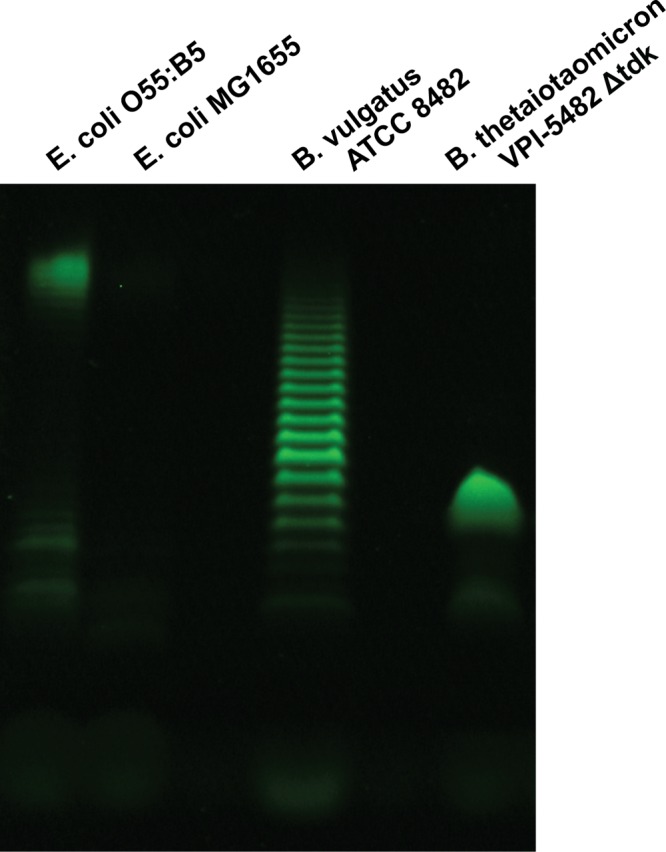
*B. thetaiotaomicron* LPS analyzed by SDS-PAGE does not appear to have an O antigen. Purified LPS from *E. coli* O55:B5, *E. coli* MG1655, *B. vulgatus* ATCC 8482, and the *B. thetaiotaomicron* Δ*tdk* mutant were run on a 16% Tricine SDS-PAGE gel. *B. vulgatus* LPS has a laddered pattern similar to that of LPS from *E. coli* O55:B5, suggesting the presence of an O-antigen repeating unit, but *B. thetaiotaomicron* LPS appears to lack a repeating unit and is therefore better characterized as lipooligosaccharide (LOS). *B. vulgatus* LPS was extracted using the microscale LPS method, and *B. thetaiotaomicron* LOS was extracted using the large-scale LPS method. LPS from *E. coli* O55:B5 and *E. coli* MG1655 was purchased from InvivoGen.

In contrast to the level of structural detail already available about *B. thetaiotaomicron* lipid A, relatively little is known about the oligosaccharide component of the *Bacteroides* LPS molecule. Purified LPS molecules can be visualized by SDS-PAGE to gain a general idea of the number of molecules present in the preparation and their relative sizes. We purified LPS from *B. thetaiotaomicron* and *B. vulgatus* ATCC 8482 (*B. vulgatus*) and compared them to commercially available *E. coli* O55:B5 and *E. coli* MG1655 LPS to confirm the previous observation that *B. vulgatus* produces LPS exhibiting a “laddered” pattern on a gel like *E. coli* O55:B5 does, whereas *B. thetaiotaomicron* LPS does not (11, 12). The laddering pattern is of note because it indicates the presence of an O antigen; the number of repeating units added to the core oligosaccharide is variable, so the result is a population of LPS molecules of different sizes. As shown in Fig. 4, *B. vulgatus* LPS appears to have an O antigen based on the laddered pattern of its LPS, but *B. thetaiotaomicron* instead appears to synthesize a small number of structures that we propose are more likely to be lipooligosaccharides (LOSs) due to their apparent lack of an O antigen. Here, we will refer to the *B. thetaiotaomicron* outer membrane glycolipid as a LOS rather than LPS.

Recent work on the human-associated bacteria *H. pylori* and *Hafnia alvei* indicates that the glycan portion of LPS from these strains can interact with C-type lectin receptors DC-SIGN and dectin-2, respectively, to influence a dendritic cell’s cytokine output (37, 38). Engagement of this class of receptors by a human-associated bacterium lacking an O antigen has not been investigated, however, and so we sought to identify the biosynthetic route for the oligosaccharide component of *B. thetaiotaomicron* LOS to explore its apparent lack of an O antigen and provide tools for manipulating the glycan structure.

### Identification of the gene cluster for LOS oligosaccharide biosynthesis in *B. thetaiotaomicron*.

Previous work analyzing biosynthetic gene clusters from the NIH Human Microbiome Project data indicated that the phylum *Bacteroidetes* contains the largest number of predicted saccharide-producing gene clusters (39). *B. thetaiotaomicron* alone is known to harbor eight gene clusters responsible for making different capsular polysaccharides (CPSs) (40, 41). We first wanted to determine whether any of these CPS gene clusters influenced the assembly of LOS. We isolated LOS from a *B. thetaiotaomicron* mutant constructed by Martens and coworkers in which all eight of its CPS clusters have been deleted (labeled the ΔCPS strain), as well as eight additional strains that each possess only one CPS cluster (CPS1-only, CPS2-only, and so on) (42). By SDS-PAGE analysis, we determined that neither deletion nor expression of CPS clusters affects the banding pattern of *B. thetaiotaomicron* LOS, indicating that these gene clusters do not encode the biosynthetic machinery for synthesis of *B. thetaiotaomicron* LOS (Fig. S2).

10.1128/mBio.02289-17.2 FIG S2  Capsular polysaccharide gene clusters do not appear to be involved in LOS biosynthesis. LOS was isolated from all 10 of these strains using the microscale extraction method and analyzed by SDS-PAGE and staining with the Pro-Q Emerald 300 lipopolysaccharide gel stain kit (Thermo Fisher). CPS bands can be seen for certain samples like CPS2-only and CPS4-only near the top of the gel. Whether the strains contain the gene clusters for all the CPSs (Δ*tdk*), none of the CPSs (ΔCPS), or one CPS at a time (CPS1-only, CPS2-only, and so on), the LOS bands appear unchanged, suggesting that the CPS gene clusters do not contribute to LOS biosynthesis. Download FIG S2, EPS file, 2.3 MB. Copyright © 2018 Jacobson et al. 2018 Jacobson et al. This content is distributed under the terms of the Creative Commons Attribution 4.0 International license.

**FIG 5:**
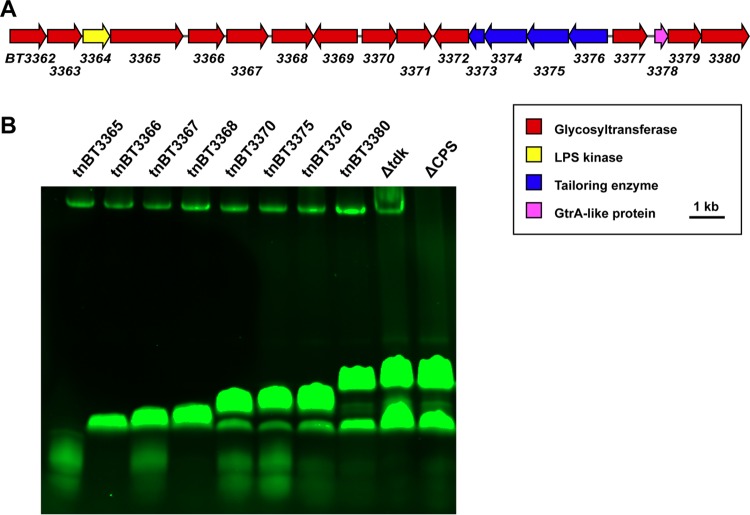
The gene cluster BT3362 to BT3380 is responsible for the biosynthesis of the *B. thetaiotaomicron* LOS oligosaccharide region. (A) Schematic of the gene cluster BT3362 to BT3380, which includes genes predicted to encode 13 glycosyltransferases shown in red, those for an LPS kinase shown in yellow, those for four tailoring enzymes shown in blue, and one for a putative GtrA-like protein shown in magenta. (B) LOS was isolated from the eight *B. thetaiotaomicron* transposon mutants, the *B. thetaiotaomicron* Δ*tdk* strain, and the *B. thetaiotaomicron* ΔCPS strain by the microscale extraction method, and the resulting material was run on a 16% Tricine SDS-PAGE gel. The transposon mutants are labeled “tn” followed by the locus tag of the gene in which the transposon has inserted. For example, “tnBT3365” indicates a *B. thetaiotaomicron* strain with a transposon inserted in BT3365. All transposon mutants are in the wild-type *Bacteroides thetaiotaomicron* VPI 5482 background with the *tdk* gene present and are erythromycin resistant. The LOS migrates on the gel differently for almost all of the transposon mutants compared to *B. thetaiotaomicron* Δ*tdk* and *B. thetaiotaomicron* ΔCPS strains; only the *B. thetaiotaomicron* tnBT3380 mutant, with the transposon inserted in the last gene in the cluster, has the same banding pattern on the gel as the two strains with wild-type LOS. The band at the top of the gel is CPS, which is present in all of the transposon mutants and the *B. thetaiotaomicron* Δ*tdk* strain but not the *B. thetaiotaomicron* ΔCPS strain.

An independent lead came from a recently published report: by screening a *B. thetaiotaomicron* transposon library using an antibody that binds the bacterial cell surface of *B. thetaiotaomicron*, Peterson et al. identified a gene cluster that they predicted might be responsible for the biosynthesis of the *B. thetaiotaomicron* LPS O antigen, due to a lack of antibody binding when the cluster was disrupted and the annotated functions of several of the genes within the cluster (43). Surprisingly, nine out of the 13 transposon mutants that did not bind the antibody had insertions in genes in the same gene cluster, BT3362 to BT3380 (Fig. 5A). No transposon insertions were obtained in the first three genes of the cluster, indicating that these genes might be essential or their deletion might lead to the accumulation of a toxic intermediate. Intrigued, we obtained a subset of these transposon mutants and analyzed LOS isolated from each mutant by SDS-PAGE (Fig. 5B). Each mutant produced LOS with a banding pattern that appeared different from that of the wild type, except for the mutant with an insertion in the final gene in the cluster, BT3380. These data suggest that BT3362 to BT3380 encode the biosynthesis of the *B. thetaiotaomicron* LOS oligosaccharide. Additionally, they support our hypothesis that bands observed on the SDS-PAGE gel are glycans that do not have a single polymerized repeating unit but rather are variants of a heterogeneous oligosaccharide.

Furthermore, the predicted function of the genes within the BT3362 to BT3380 cluster also supports this conclusion. This cluster bears some resemblance to the *waa* core oligosaccharide gene clusters characterized in *E. coli*, with the first gene, BT3362, sharing homology with the genes for heptosyltransferases WaaC and WaaQ (44). Overall, the cluster possesses 13 predicted glycosyltransferases (BT3362-BT3363, BT3365 to BT3372, BT3377, and BT3379-BT3380), a putative LPS kinase (BT3363), four tailoring enzymes (BT3373 to BT3376), and a GtrA-like protein (BT3378). It is unclear what specific structural modifications BT3373 to BT3376 might make based solely on sequence homology. Proteins in the GtrA-like family are typically integral membrane proteins that are thought to play a role in the transport of cell surface polysaccharides (45, 46).

### Intact LPS MALDI-TOF MS as a diagnostic tool for LOS mutants.

While the SDS-PAGE analysis of LOS from the transposon mutants implicates BT3362 to BT3380 in *B. thetaiotaomicron* LOS oligosaccharide biosynthesis, we wanted to increase the resolution of our analysis using mass spectrometry (MS). The LPS/LOS bands on an SDS-PAGE gel are approximations of the sizes of molecules in a sample, and using mass spectrometry would allow us to gain a clearer picture of the molecules made by the transposon mutants. Although it is easier to analyze LPS/LOS by MALDI-TOF after removing the O- and/or N-linked acyl chains via hydrazine or hydrogen fluoride treatment, we chose to analyze intact LOS molecules that were not subjected to chemical degradation or derivatization. We reasoned that LOS from *B. thetaiotaomicron* may contain important functional groups in the oligosaccharide chain that could be removed by these treatments, further complicating our efforts to elucidate detailed structural information about *B. thetaiotaomicron* LOS.

**FIG 6:**
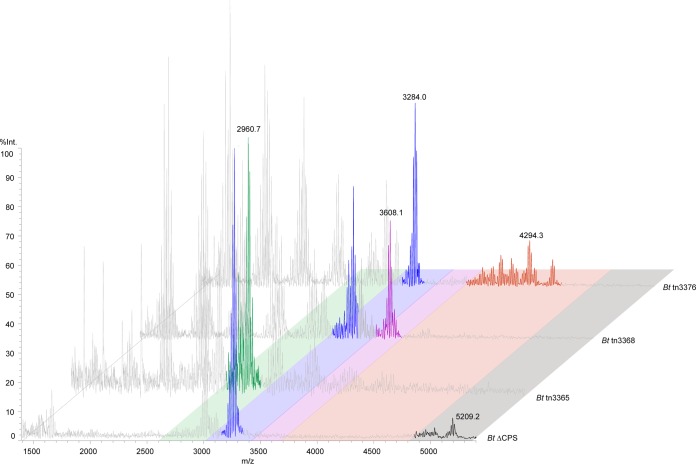
MALDI-TOF MS analysis of intact LOS indicates that the transposon mutants make truncated LOS compared to wild type. LOSs from *B. thetaiotaomicron* ΔCPS, *B. thetaiotaomicron* tnBT3365, *B. thetaiotaomicron* tnBT3368, and *B. thetaiotaomicron* tnBT3376 strains were isolated using the large-scale LPS/LOS extraction method. The resulting material was desalted and spotted on a THAP-nitrocellulose matrix for analysis on a Shimadzu Axima Performance MALDI-TOF mass spectrometer in linear negative-ion mode. LOS peaks of interest have been colored, with each color representing a different LOS species present in the different strains. Peaks colored gray are likely derived from capsular polysaccharide (those that do not appear in the *B. thetaiotaomicron* ΔCPS spectrum) or lipid A in the case of the peak clusters around 1,650 to 1,700 *m/z*.

We adapted a previously published strategy for analyzing intact LPS/LOS by MALDI-TOF (47, 48). LPS/LOS analysis is typically challenging due to difficulties in inducing the glycolipid to ionize because of its size and polarity. We chose three transposon mutants that appeared by SDS-PAGE analysis to be truncated to various degrees (*B. thetaiotaomicron* tn3365, *B. thetaiotaomicron* tn3368, and *B. thetaiotaomicron* tn3376), along with LOS isolated from the *B. thetaiotaomicron* ΔCPS strain. The ΔCPS strain has wild-type LOS biosynthetic genes, but its lack of CPS yielded mass spectra with a cleaner background. The transposon mutants were created in the background of wild-type *B. thetaiotaomicron*, and so CPS is present in those preparations. In LOS from the ΔCPS strain, we observed a cluster of peaks around 5,209 *m/z*, the largest mass detected for any of the samples (Fig. 6). Although instrument-specific limitations prevented us from obtaining a resolution as high as others have observed, the limited degree of resolution that we achieved was sufficient for confirming the approximate masses of LOS molecules in the sample and comparing them to those of the truncated mutants (8, 48).

In addition to the peak that we propose corresponds to full-length LOS at 5,209 *m/z*, the ΔCPS sample has additional peaks at 3,284 and 3,017 *m/z*, which are likely intermediate species created by partial completion of the biosynthetic pathway. The most truncated transposon mutant in our set, tn3365, has a single predominant peak at 2,961 *m/z*. LOS from tn3368 does not have this 2,961 *m/z* peak but instead has peaks at 3,284 *m/z* (as in the ΔCPS sample) and 3,608 *m/z*. Interestingly, 2,961, 3,284, and 3,608 *m/z* are separated from one another by ~324 Daltons. The expected mass of a hexose is ~162 Daltons, and so we predict that tn3368 makes two LOS molecules that are two and four hexose units longer than the molecule made by tn3365. Finally, LOS from the least truncated transposon mutant that we assayed, tn3376, has its largest peaks around 4,497 and 4,295 *m/z*, as well as the 3,284 *m/z* peak that is common to both the tn3368 and the ΔCPS samples. The mass differences between tn3368 and tn3376, as well as between tn3376 and ΔCPS, do not suggest a structural difference as straightforward as the addition of hexoses between tn3365 and tn3368. Given the presence of genes encoding tailoring enzymes in the LOS biosynthetic cluster, we expect that the longer LOS species have modifications like phosphorylation, acetylation, or carbamoylation. All of the transposon mutants have additional peaks between 1,500 and 3,000 *m/z* that presumably derive from CPS, since these peaks are absent in the ΔCPS sample but present in LOS isolated from the *B. thetaiotaomicron Δtdk* mutant (Fig. S3). Additionally, every sample has a cluster of peaks around 1,688 *m/z* representing lipid A, which likely derives from in-source fragmentation (48).

10.1128/mBio.02289-17.3 FIG S3  Capsular polysaccharide is likely the cause of contaminating peaks in samples in the *Δtdk* wild-type background. LOS from the *B. thetaiotaomicron Δtdk* strain was purified by the large-scale extraction method, and the intact molecule was analyzed on a Shimadzu Axima Performance MALDI-TOF MS instrument in linear negative-ion mode. The peaks in black are those species that are also present in the ΔCPS intact LOS sample, but the rest of the peaks in red are unique to the *Δtdk* sample despite its differing from the ΔCPS mutant only in that it contains capsule. Because similar peaks are noted in the intact LOS MALDI-TOF spectra of the transposon mutants, which also contain capsule, we conclude that the peaks in red are due to the presence of CPS in the LOS preparation. Download FIG S3, EPS file, 1.6 MB. Copyright © 2018 Jacobson et al. 2018 Jacobson et al. This content is distributed under the terms of the Creative Commons Attribution 4.0 International license.

With the goal of confirming these results using clean deletion mutants rather than the transposon mutants, we deleted as much of the cluster as we could—BT3363 and BT3365 to BT3380 (we could not obtain mutants of BT3362 and BT3364, consistent with the lack of transposon insertions in these genes) (43). Surprisingly, when we compared LOSs purified from this strain and from the tn3365 mutant on an SDS-PAGE gel, the banding pattern of *B. thetaiotaomicron* ΔBT3363 ΔBT3365–BT3380 LOS appeared to resemble that of wild-type LOS. However, when we subjected the *B. thetaiotaomicron* ΔBT3365 ΔBT3365–BT3380 LOS to MALDI-TOF analysis, the mass of the intact glycolipid was around 4,870 Daltons, smaller than the 5,209 Daltons seen in wild-type LOS (Fig. S4). These data suggest that there might be a compensatory mechanism in which the bacterium is able to glycosylate truncated forms of the LOS molecule. Peterson et al. similarly predicted that *B. thetaiotaomicron* may have the ability to produce an O antigen when this gene cluster is disrupted because the colony morphology of their transposon mutants did not differ from that of wild-type *B. thetaiotaomicron* (43). However, by our intact LOS MALDI-TOF analysis, we see evidence for new oligosaccharide production only when the cluster is cleanly deleted, rather than when single genes are disrupted by transposon insertion. Our result highlights the limitations of SDS-PAGE analysis in determining structural differences between LOS samples (Fig. S5). Further studies need to be conducted to understand whether *B. thetaiotaomicron* has an alternative lipid A glycosylation pathway that is unmasked in the absence of most of the LOS oligosaccharide gene cluster.

10.1128/mBio.02289-17.4 FIG S4  *B. thetaiotaomicron* produces a molecule with a different mass when the LOS oligosaccharide biosynthesis cluster is deleted. LOS was isolated from the *B. thetaiotaomicron* ΔCPS ΔBT3363 ΔBT3365–BT3380 (red) and *B. thetaiotaomicron* ΔBT3363 ΔBT3365–BT3380 (black) strains by the large-scale extraction method and analyzed on a Shimadzu Axima Performance MALDI-TOF MS instrument in linear negative-ion mode. These mutants have as much of the LOS oligosaccharide biosynthesis cluster deleted as possible and should not be able to make glycosylated LOS, and yet they appear to make new molecules in its stead. Lipid A is still present in both samples, suggesting that a new oligosaccharide may be getting attached to the truncated LOS left over after the cluster deletion. Download FIG S4, EPS file, 1.7 MB. Copyright © 2018 Jacobson et al. 2018 Jacobson et al. This content is distributed under the terms of the Creative Commons Attribution 4.0 International license.

10.1128/mBio.02289-17.5 FIG S5  LOSs from a transposon insertion strain and a clean deletion strain do not have the same banding pattern on an LOS gel. LOS was isolated from *B. thetaiotaomicron* ΔBT3365 and *B. thetaiotaomicron* tnBT3365 strains by the large-scale LPS/LOS extraction method. The resulting material was analyzed by SDS-PAGE and stained using the Pro-Q Emerald 300 lipopolysaccharide gel stain kit (Thermo Fisher). While a transposon insertion in BT3365 results in what appears to be a truncated LOS structure, a clean deletion of the same gene looks like it has a wild-type banding pattern. Download FIG S5, EPS file, 0.5 MB. Copyright © 2018 Jacobson et al. 2018 Jacobson et al. This content is distributed under the terms of the Creative Commons Attribution 4.0 International license.

### Predicting other *Bacteroides* LOS oligosaccharide gene clusters.

**FIG 7:**
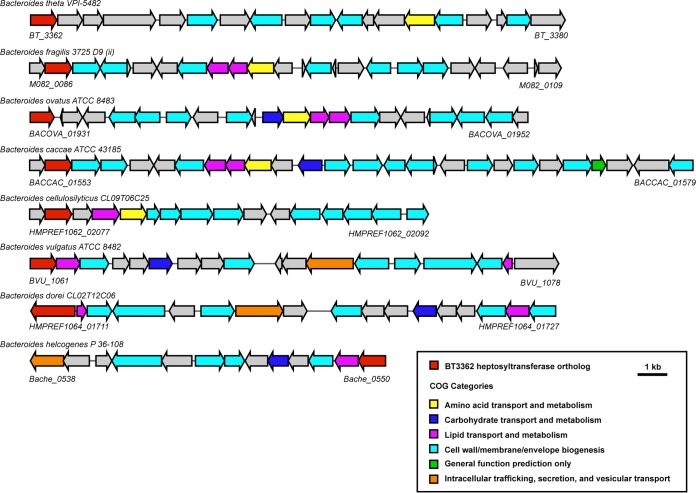
Clusters orthologous to the *B. thetaiotaomicron* LOS oligosaccharide gene cluster in additional *Bacteroides* species. Orthologs of the BT3362 to BT3380 cluster in *B. thetaiotaomicron* were identified using the “Show neighborhood ortholog regions with the same top COG hit” function within the DOE Joint Genome Institute’s IMG portal, using BT3362, the putative *B. thetaiotaomicron* LOS heptosyltransferase, as the query gene. We have estimated the start and end of each gene cluster by inspecting the surrounding genomic area of each ortholog and comparing it to the *B. thetaiotaomicron* cluster. Our list shows a representative sample of the clusters resulting from this search.

Having identified the probable *B. thetaiotaomicron* LOS biosynthetic gene cluster, we hypothesized that it could be used to identify candidate LOS and LPS gene clusters in other *Bacteroides* species. We used the two essential genes in the cluster, BT3362 (a putative heptosyltransferase) and BT3364 (a putative LPS kinase), as queries in BLAST searches against other *Bacteroides* genomes and were able to identify similar clusters in many *Bacteroides* species (Fig. 7). Given that a homologous cluster is found in *B. vulgatus*, which elaborates a laddered LPS, we expect that *B. vulgatus* harbors an additional cluster encoding the biosynthesis of the O antigen repeating unit. This would likely be attached to the product of the *B. thetaiotaomicron-*like core oligosaccharide.

Our results are a first step in characterizing what we expect will be a large amount of biosynthetic and structural heterogeneity among *Bacteroides* LPS or LOS molecules. Given our understanding of the privileged role that glycolipids play in communicating with the mammalian immune system and the sheer quantity of LPS in the gut, *Bacteroides* LPS/LOS molecules are likely to be critical mediators in the interaction between commensal microbes and the host. By understanding how these molecules are made, we gain the possibility of manipulating their structure and by extension the host’s immune response.

## MATERIALS AND METHODS

### Bacterial strains, growth conditions, and reagents.

See Table S1 in the supplemental material for a full list of bacterial strains and plasmids used in this study. All *Bacteroides* strains were cultured anaerobically at 37°C in peptone-yeast extract-glucose (PYG) liquid medium or brain heart infusion (BHI) agar (BD Biosciences) with defibrinated horse blood (Hardy Diagnostics) added to 10% vol/vol. The *B. thetaiotaomicron* transposon mutants were grown in liquid and agar media supplemented with 25 μg/ml erythromycin. *B. thetaiotaomicron* strains needed for large-scale LPS extraction were grown in BHI broth (BD Biosciences) supplemented with 5 μg/ml hemin and 500 μg/ml l-cysteine hydrochloride (Sigma-Aldrich). The gas mix for the anaerobic chamber (Coy Laboratory Products) was 5% hydrogen and 20% carbon dioxide, balanced with nitrogen (Airgas). The *E. coli* strains used for cloning the pExchange-*tdk* knockout constructs were cultured in Luria broth (LB) or agar supplemented with carbenicillin. LPS from *E. coli* O55:B5 and *E. coli* MG1655 was purchased from InvivoGen (LPS-B5 Ultrapure and LPS-K12 Ultrapure, respectively).

10.1128/mBio.02289-17.6 TABLE S1  Strains, plasmids, and primers used in this study. (A) List of strains and plasmids. (B) List of primers and their sequences used for cloning and sequencing. Download TABLE S1, EPS file, 1 MB. Copyright © 2018 Jacobson et al. 2018 Jacobson et al. This content is distributed under the terms of the Creative Commons Attribution 4.0 International license.

### Construction of *B. thetaiotaomicron* clean deletion mutants.

See Table S1 for a list of all primers used for making scarless gene deletions in *B. thetaiotaomicron*. To make clean deletions as described by Koropatkin et al., approximately 1,000 bp upstream and downstream of the desired gene to be deleted, including the start and stop codons, respectively, were amplified by PCR (49). For BT2152 and BT1854, the two fragments were stitched together by PCR, taking advantage of the overlapping regions in the original primers. For BT2152, the pExchange-*tdk* vector (gift from Justin Sonnenburg, Stanford University) and the fused insert were digested with SalI and NotI and ligated together. For BT1854, only the pExchange-*tdk* vector was digested with SalI and XbaI, and the insert was assembled with the vector by circular polymerase extension cloning (CPEC). For BT3363, BT3365, and BT3365 to BT3380, Gibson assembly was used to combine the upstream and downstream fragments with SpeI/NotI-digested pExchange-*tdk*. Ligation, CPEC, and Gibson assembly products were transformed into electrocompetent *E. coli* S17-1 *λ pir* (Bio-Rad MicroPulser; 18 kV in 0.1-cm cuvettes), selected for on LB agar plus carbenicillin, and conjugated into *B. thetaiotaomicron* VPI 5482 *Δtdk*, which serves as the background strain for all the clean deletions. Single recombinants were selected for on BHI-blood agar supplemented with 200 μg/ml gentamicin and 25 μg/ml erythromycin, picked, cultured overnight in PYG medium, and plated on BHI-blood agar supplemented with 200 μg/ml 5-fluoro-2-deoxyuridine. Colonies were screened for success of the gene deletion by PCR, and deletion was confirmed by DNA sequencing.

### The TRI reagent method for lipid A extraction.

To purify lipid A from *Bacteroides* species, we used the TRI reagent method (24). Briefly, bacterial cells were grown in 10-ml liquid cultures to mid-log phase, harvested by centrifugation at 3,270 × *g* for 10 min, resuspended in 1 ml TRI reagent (Molecular Research Center), vortexed vigorously, and left at room temperature for 10 min. One milliliter of chloroform was added, and the mixture was vortexed and left at room temperature for another 10 min. The mixture was centrifuged at 15,000 × *g* for 10 min, and the aqueous layer was removed to a clean tube. The sample was extracted again with 200 μl deionized water, and the resulting aqueous layer was removed to the same tube as described above. The aqueous extraction was repeated two more times, and the collected aqueous material was pooled and lyophilized (Labconco FreeZone). A mild acid hydrolysis was used to separate the lipid A from its poly- or oligosaccharide chain. The lyophilized material was resuspended in 1 ml 10 mM sodium acetate (pH 4.5) and 1% sodium dodecyl sulfate (SDS). Samples were boiled at 100°C for 1 h and then lyophilized again. To remove SDS from the lipid A, the lyophilized material was washed with 1 ml ice-cold acidified ethanol (20 mM hydrochloric acid in 95% ethanol) once followed by three washes each with 500 μl ice-cold 95% ethanol, centrifuging the mixture at 3,270 × *g* at 4°C for 5 min each time. A Bligh-Dyer extraction was used to separate the hydrolyzed lipid A from the saccharides, and the organic and interface layers were removed to a small glass vial. Solvent was removed with a rotary evaporator, and the material was stored at −20°C.

### MALDI-TOF mass spectrometry analysis of lipid A. (i) Sample and matrix preparation.

Three to five drops of 3:1 chloroform-methanol was added to a thawed vial of dried lipid A, with gentle rocking to help the lipid A dissolve. The matrix was a saturated solution of 5-chloro-2-mercaptobenzothiazole (CMBT) in 3:1 chloroform-methanol. To spot the sample on the target, 3 μl of dissolved sample was mixed with 3 μl matrix, and 1 μl of the resulting mixture was spotted within an inscribed circle on the MALDI target (Waters Corporation) and allowed to dry (50).

### (ii) Negative-ion MALDI-TOF MS.

MALDI-TOF MS analysis was carried out on a Waters Corporation Synapt G2 high-definition mass spectrometer with a 355-nm neodymium-doped yttrium aluminum garnet (Nd:YAG) laser in reflectron negative-ion mode. The instrument was calibrated using a mixture of angiotensin II, renin substrate, insulin chain B, and bovine insulin (Sigma-Aldrich), with monoisotopic [M-H]^−^ ion masses of 1,044.5267, 1,756.9175, 3,492.6357, and 5,728.5931 *m/z*, respectively (51). The standards were dissolved together in a solution of 0.1% trifluoroacetic acid in water. Because the standard mixture was not in the same solvent as the CMBT matrix mentioned above, 1 μl of CMBT matrix was spotted on the target and allowed to dry before 1 μl of the standard mixture was spotted on top of the matrix. MS data were collected between 400 and 5,000 *m/z*, and the resulting spectra were smoothed and baseline corrected using MassLynx software.

### Large-scale LPS/LOS extraction.

LPS or LOS was isolated from whole bacteria using the hot phenol-water method (52). Briefly, bacteria were grown overnight in a 10-ml culture and then expanded to 2 liters. Cells were harvested once cultures reached an optical density (OD) of at least 0.7 and pelleted by centrifugation at 6,000 × *g* for 30 min at 4°C. The entire wet cell pellet from the 2-liter culture was suspended in 20 ml water. Separately, the cell suspension and 20 ml of 90% phenol solution in water were each brought up to 68°C with stirring. Once at temperature, the phenol solution was slowly added to the cell suspension. The mixture was stirred vigorously for 30 min at 68°C and then cooled rapidly in an ice water bath for 10 min. The sample was centrifuged at 15,000 × *g* for 45 min, and the upper aqueous layer was transferred into 1,000-molecular-weight-cutoff (MWCO) dialysis tubing. The sample was dialyzed against 4 liters of water for 4 days, changing the water twice per day. LPS/LOS was pelleted out of the dialysate by ultracentrifugation at 105,000 × *g* for 4 h. The pellet was resuspended in water and treated with RNase A (Thermo Fisher), DNase I (New England Biolabs), and proteinase K (Thermo Fisher) before repeating the ultracentrifugation step. The pellet was resuspended in water, lyophilized, and stored at −20°C. LPS/LOS samples that were prepared by this method include the *B. thetaiotaomicron Δtdk* strain in Fig. 4, all samples in Fig. 6, and all samples in Fig. S3, S4, and S5.

### Microscale LPS/LOS extraction.

The microscale LPS/LOS extraction was used when a large number of samples was needed for SDS-PAGE analysis. In this method, adapted from the work of Marolda and coworkers, bacteria were grown to mid-log phase in 5 ml of medium and pelleted (53). Cell pellets were resuspended in 150 μl lysis buffer (0.5 M Tris-hydrochloride, pH 6.8, 2% SDS, 4% β-mercaptoethanol) and boiled at 100°C for 10 min. Proteinase K was added to each sample before incubation at 60°C for 1 h. The sample temperature was raised to 70°C, and 150 μl prewarmed 90% phenol in water was added. Samples were vortexed three times at 5-min intervals during a 15-min incubation. The samples were immediately cooled on ice for 10 min and centrifuged at 10,000 × *g* for 1 min. The aqueous layer (~100 μl) was pipetted into a clean tube, and 5 volumes of ethyl ether saturated with 10 mM Tris-hydrochloride (pH 8.0) and 1 mM EDTA was added. The samples were vortexed and centrifuged, and the aqueous layer was removed to a clean tube. An appropriate amount of 3× loading dye (0.187 M Tris-hydrochloride, pH 6.8, 6% SDS, 30% glycerol, 0.03% bromophenol blue, 15% β-mercaptoethanol) was added, and the samples were stored at −20°C. LPS/LOS samples that were prepared by this method include *B. vulgatus* in Fig. 4, all samples in Fig. 5, and all samples in Fig. S2.

### SDS-PAGE analysis of LPS.

To visualize LPS/LOS on an SDS-PAGE gel, we used Novex 16% Tricine protein gels (1.0 mm, 12 wells) and 10× Novex Tricine SDS running buffer (Thermo Fisher) (12). For samples prepared by the LPS/LOS microscale extraction, 15 μl of the resulting aqueous layer mixed with 3× loading dye was added to each lane. For samples prepared by LPS/LOS large-scale extraction or purchased from InvivoGen, 2.5 μg of material was resuspended in 15 μl 1× loading dye and the whole volume was added to a lane. Gels were run at 125 V for 90 min at room temperature, stained with Pro-Q Emerald 300 lipopolysaccharide gel stain (Thermo Fisher) per the manufacturer’s instructions, and imaged on a Bio-Rad Gel Doc EZ Imager using the SYBR green filter.

### MALDI-TOF mass spectrometry analysis of intact LOS. (i) Sample and matrix preparation.

To detect intact LPS by MALDI-TOF MS, we closely followed the technique developed by Phillips et al. adapted to study *Neisseria* lipooligosaccharides (48). One milligram of lyophilized LOS was dissolved in 100 μl 1:3 methanol-water with 5 mM EDTA. Cation exchange beads (Dowex 50WX8, 200 to 400 mesh) were converted to the ammonium form and deposited into 1.5-ml tubes before desalting the LOS. Each sample suspension was added to the beads, vortexed, and centrifuged briefly to pellet the beads. The sample was removed to a clean tube and mixed 9:1 with 100 mM dibasic ammonium citrate before spotting on the target. The matrix was made by mixing a 15-mg/ml solution of nitrocellulose membrane in 1:1 isopropanol-acetone with a 200-mg/ml solution of 2′,4′,6′-trihydroxyacetophenone (THAP) in methanol in a 1:3 ratio. The matrix was deposited by pipetting 1 μl within an inscribed circle on the target (Shimadzu) and allowed to dry. Once the matrix had dried completely, 1 μl of the sample preparation was added on top of the matrix and allowed to dry.

### (ii) Negative-ion MALDI-TOF MS.

MALDI-TOF MS analysis was performed on a Shimadzu Axima Performance mass spectrometer with an N_2_ laser in linear negative-ion mode. It was calibrated using the same solution of four standards that was used to calibrate the Waters Synapt G2 for lipid A analysis—angiotensin II, renin substrate, insulin chain B, and bovine insulin in 0.1% trifluoroacetic acid—and the standards were spotted as described above except on the THAP-nitrocellulose matrix. MS data were collected between 700 and 7,000 m*/z*, and the resulting spectra were smoothed and baseline corrected using Shimadzu Biotech Launchpad software.
